# Qualitative risk assessment of Japanese encephalitis virus introduction and transmission in Pacific Island countries and territories

**DOI:** 10.1371/journal.pntd.0014162

**Published:** 2026-09-03

**Authors:** Eloise B. Skinner, Amanda K. Murphy, Fan Yu, Kevin Thabo Moore, Liuyi Chen-Cao, David T. Williams, Stephan Karl, Joelyn Goi, Colleen L. Lau, Benn Sartorius

**Affiliations:** 1 Frazer Institute, Faculty of Health, Medicine and Behavioural Sciences, University of Queensland, Brisbane, Queensland, Australia; 2 Australian Institute of Tropical Health and Medicine, James Cook University, Smithfield, Queensland, Australia; 3 Mosquito Control Lab, QIMR-Berghofer Medical Research Institute, Brisbane, Queensland, Australia; 4 Australian Centre for Disease Preparedness, Commonwealth Scientific and Industrial Research Organisation, Geelong, Victoria, Australia; 5 Vector-borne Diseases Unit, Papua New Guinea Institute of Medical Research, Madang, Papua New Guinea; 6 Department of Health Metrics Sciences, Institute for Health Metrics and Evaluation, School of Medicine, University of Washington, Seattle, Washington, United States of America; 7 Centre for Tropical Medicine and Global Health, Nuffield Department of Population Health, University of Oxford, Oxfordshire, United Kingdom; Colorado State University, UNITED STATES OF AMERICA

## Abstract

**Background:**

The emergence of Japanese encephalitis virus (JEV) in Australia raises questions about transmission potential across neighbouring Pacific Island countries and territories (PICs), where surveillance infrastructure and diagnostic capacity are limited.

**Methodology:**

We conducted a qualitative risk assessment of 22 PICs structured around three components: (1) ‘release assessment’, evaluating JEV introduction pathways based on geographic proximity, migratory waterbird flyway connectivity, and windborne mosquito dispersal potential; (2) ‘exposure assessment’, evaluating ecological transmission suitability based on vector and host competence and occurrence; and (3) ‘risk characterisation’, integrating release and exposure components to stratify countries into transmission risk profiles (endemic, epidemic, and low potential). Introduction potential varied across the region, with northern and western PICs having greatest connectivity to endemic regions. Ecological suitability for transmission was sufficient to support JEV transmission in 13 of the 22 PICs if introduced. Eight PICs were characterised as having endemic transmission potential (Papua New Guinea, which already has confirmed circulation, Solomon Islands, New Caledonia, Vanuatu, Fiji, Palau, Guam, and Northern Mariana Islands), based on diverse competent vector communities, high ardeid waterbird diversity, and moderate-to-high introduction potential. Five PICs (Federated States of Micronesia, Tonga, Samoa, Tuvalu, and Nauru) were characterised as having epidemic transmission potential, supported by pig densities capable of amplifying viral titres and sufficient vector diversity, but lacking ardeid populations necessary to sustain endemic cycles. The remaining nine PICs had low transmission potential due to limited vector and host communities. These risk stratifications are relative; the absolute probability of JEV establishment in any PIC remains uncertain.

**Conclusions:**

Distinct transmission profiles require differentiated public health responses, from sustained surveillance in endemic-potential settings to early detection and rapid outbreak response in epidemic-potential settings. Regional coordination through shared diagnostic networks and vaccine access pathways is critical to support preparedness before JEV becomes established across the Pacific.

## Author summary

Japanese encephalitis virus is a mosquito-borne pathogen that causes severe brain inflammation and is responsible for significant illness and death across Asia. Whether this virus poses a threat to Pacific Island nations has received little attention, despite the proximity of some islands to regions where the virus circulates. We assessed the potential for Japanese encephalitis virus introduction and transmission across 22 Pacific Island countries and territories by examining existing data on mosquito species, waterbird and pig populations, and connectivity to endemic regions through migratory bird flyways and geographic proximity.

We found that most Pacific Island countries and territories have ecological conditions that could support transmission if the virus were introduced. This work represents a first attempt to systematically evaluate Japanese encephalitis virus risk across the Pacific using available data. Our findings suggest that strengthening surveillance capacity and regional coordination now, before the virus becomes established, would help health systems prepare for a threat that may be closer than previously recognised.

## Introduction

Japanese encephalitis virus (JEV) is a mosquito-borne flavivirus (*Orthoflavivirus japonicum*) and the leading cause of viral encephalitis in Asia, with an estimated 68,000–100,000 cases annually [1]. Most infections are asymptomatic, but when clinical disease occurs, typically in fewer than 1% of infections [2], it can cause severe neurological illness including high fever, seizures, coma, and death, with a 20–30% case fatality rate and long-term disability in up to 50% of survivors [1–3]. JEV is a vaccine-preventable disease, with safe and effective human vaccines available, making early identification of high-risk settings essential for guiding timely public health interventions and vaccination strategies [4]. JEV is also an important pathogen of livestock animals, causing potentially fatal encephalitis in horses and reproductive disease in pigs, leading to abortion and neurological disease in piglets [5,6]. In Pacific Island communities, particularly Melanesia, pigs hold cultural and economic significance beyond their role as a food source [7]. In these settings JEV transmission could therefore have substantial socioeconomic consequences extending beyond direct human health impacts.

JEV is classified into five genotypes (GI to GV) based on phylogenetic differences in the envelope gene, with genotypes differing in geographic distribution, transmission patterns, and antigenic properties that may influence epidemic potential and vaccine performance [8]. Historically endemic in South, Southeast, and East Asia, JEV has demonstrated a capacity for geographic range expansion in recent decades. Genotype I has progressively replaced genotype III as the dominant circulating strain across much of Asia, while genotype V has re-emerged in China and South Korea after decades of apparent absence [8–10]. Genotype IV emerged in northern parts of mainland Australia in 2021 [11] before spreading to temperate regions of southeastern Australia in the 2021–22 summer, causing human and animal cases and establishing transmission cycles in areas previously considered outside the virus’s climatic range [8,12]. Subsequent human cases in Australia during 2024 and 2025 have confirmed this is not a transient incursion, raising urgent questions about transmission potential across neighbouring Pacific Island countries and territories (PICs) and areas that may share similar ecological conditions.

Geographic expansion of JEV into new regions can occur through multiple mechanisms. Migratory waterbirds, particularly ardeid species, can transport the virus along established flyways connecting endemic Asia to the Pacific and Australasia during their seasonal movements [13,14]. Windborne dispersal of infected mosquitoes represents another potential pathway, with passive aerial transport of mosquitoes over distances of tens to hundreds of kilometres now confirmed through high-altitude sampling studies [15,16]. Modelling studies specific to the Australasian region support this and found that mosquitoes could be transported over water for distances of 200–300 km during favourable meteorological conditions, providing a plausible mechanism for JEV introduction from Papua New Guinea to northern Australia [17]. Finally, incursion of exotic mosquito species via international cargo and air travel represents an additional pathway, with stowaways in shipping containers, aircraft holds, and the used tyre trade well documented as mechanisms for establishment of invasive vector species in new regions [18]. Understanding introduction mechanisms is essential for assessing JEV incursion risks and for prioritising surveillance efforts accordingly.

JEV is maintained through complex vector–host transmission cycles that involve interactions between competent *Culex* mosquito species and vertebrate hosts capable of viral replication and transmission [19]. Foundational studies in the 1950s established that ardeid waterbirds serve as reservoir hosts capable of sustaining long-term viral circulation, while domestic pigs function as amplifying hosts that generate high viremia levels [20]. While there is increasing evidence that species other than pigs and waterbirds, such as sheep [21] and bats [22], may contribute to JEV transmission cycles, the extent to which they do is still under consideration. Humans and many other vertebrates (including cattle, horses, goats, dogs, and chickens) are generally considered dead-end hosts because infection does not result in viremia levels sufficient to transmit the virus [19,23]. The different roles vertebrate species play (e.g., reservoir, amplifier, dead-end host) can result in distinct disease ecology profiles across the geographic distribution of JEV. Endemic JEV transmission, defined here as sustained viral circulation through enzootic cycles between wildlife reservoir hosts (i.e., waterbirds) and mosquito vectors, can allow JEV to persist. While epidemic JEV transmission, defined here as capacity for rapid amplification in domestic pig populations, can generate substantial outbreak activity but typically results in self-limiting transmission due to the removal of infected pigs, and lack of wildlife reservoirs to sustain ongoing transmission. Different transmission profiles such as these are important to consider when assessing the risk of JEV in an area because they can determine whether control measures should focus on preventing establishment of long-term endemic cycles or on rapid outbreak detection and response to limit human and animal cases during episodic introductions.

Formal disease risk assessment frameworks provide systematic approaches for evaluating the probability and consequences of pathogen introduction and establishment in new geographic areas. Established frameworks typically incorporate ‘release assessment’ (probability of pathogen entry), ‘exposure assessment’ (likelihood of susceptible populations encountering the pathogen), ‘consequence assessment’ (magnitude of health and economic impacts), and ‘risk characterisation’ that integrates these components [24,25]. Quantitative risk assessments produce probability estimates for specific outcomes, while qualitative and semi-quantitative approaches use categorical rankings when empirical data are limited [24,26,27]. In settings where pathogens have not yet established and surveillance data are absent or minimal, probabilistic risk estimates face substantial uncertainty due to limited baseline information on vector infection rates, host population dynamics, and actual introduction frequencies through different pathways. Under these conditions, structured qualitative assessment approaches that integrate available ecological and epidemiological data through transparent criteria can provide actionable risk stratification to guide public health preparedness, even when precise probability estimates cannot be generated [27]. For PICs, most of which lack active JEV surveillance programs and, apart from Papua New Guinea (PNG), have no documented transmission history, such systematic assessment methods are appropriate where ecological conditions and introduction pathways vary substantially.

The objective of this research is to conduct a qualitative risk assessment examining release (introduction pathways), exposure (ecological suitability), and risk characterisation to stratify JEV transmission profiles across 22 PICs. Using a multi-criteria assessment approach with explicit scoring thresholds, we aim to distinguish settings where endemic cycles could establish, where epidemic transmission might occur without long-term persistence, and where ecological constraints limit transmission potential.

## Methods

This study applied qualitative risk assessment methods to evaluate JEV introduction and transmission potential across all 22 PICs (American Samoa, Cook Islands, Federated States of Micronesia, Fiji, French Polynesia, Guam, Kiribati, Marshall Islands, Nauru, New Caledonia, Niue, Northern Mariana Islands, Palau, Papua New Guinea, Pitcairn Islands, Samoa, Solomon Islands, Tokelau, Tonga, Tuvalu, Vanuatu, and Wallis and Futuna). Following established frameworks for animal and human health risk assessments [24,25,27,28], we structured our assessment around three core components: (1) release assessment, evaluating pathways and potential for JEV introduction from endemic regions; (2) exposure assessment, evaluating ecological suitability for JEV transmission based on vector presence and competent host populations; and (3) risk characterisation, integrating these components to stratify countries according to their capacity to support endemic or epidemic JEV transmission. This multi-criteria qualitative approach uses categorical scoring with explicit thresholds to provide transparent risk stratification in the absence of direct surveillance data [27].

### Baseline JEV status

To establish the baseline status of JEV in the PICs, a scoping literature review was conducted with the aim to identify any evidence of historic or current JEV circulation in any of the 22 countries or territories. Searches were performed in PubMed, Web of Science and Google Scholar using terms including (“Japanese encephalitis” OR “JEV” OR “Japanese encephalitis virus”) AND (“Pacific” OR “PICT” OR “Pacific Island” OR “Melanesia” OR “Micronesia” OR “Polynesia” OR “American Samoa” OR “Cook Islands” OR “Federated States of Micronesia” OR “Fiji” OR “French Polynesia” OR “Guam” OR “Kiribati” OR “Marshall Islands” OR “Nauru” OR “New Caledonia” OR “Niue” OR “Northern Mariana Islands” OR “Palau” OR “Papua New Guinea” OR “Pitcairn Islands” OR “Samoa” OR “Solomon Islands” OR “Tokelau” OR “Tonga” OR “Tuvalu” OR “Vanuatu” OR “Wallis and Futuna”) from 1900 to October 2025. Additional targeted searches used “seroprevalence” OR “surveillance” OR “mosquito pool” OR “virus isolation”. Reference lists of relevant reviews and reports (e.g., WHO, CDC, Pacific Public Health Surveillance Network) were hand-searched. For papers reporting evidence of JEV circulation, we extracted information on the location, timing of detection, number of human cases reported, and additional epidemiological data including serological surveys, entomological surveillance findings, and evidence from sentinel animal or wildlife populations.

### Release assessment: Introduction pathways

The release assessment, which evaluates introduction pathways, assessed the potential of JEV incursion based on two factors; 1) the geographic proximity to endemic countries with consideration for wind-blown mosquito dispersal, and 2) overlap with migratory waterbird flyways which can connect Pacific islands to JEV-endemic areas of Asia. While JEV introduction via infected mosquitoes transported in commercial aircraft or cargo vessels and/or movement of infected livestock between regions remains theoretically possible, we considered these pathways to be of lower probability compared to natural dispersal mechanisms and did not include them in our formal risk scoring. Distances from each PIC to the nearest JEV-endemic country were measured as the shortest coast-to-coast distance using Google Earth Engine. Flyway boundaries were obtained from BirdLife International’s DataZone [29]. Countries were classified as high introduction risk if located within the East Asian-Australasian Flyway and within 1,000km of JEV-endemic countries, moderate risk if either criterion were met, and low risk if both criteria were not met (Table 1).

**Table 1:** Classification thresholds for JEV risk assessment components.

Component	Low	Moderate	High
Vector suitability (number of competent/probable species)	<5	5-9	≥10
Ardeid host suitability (confirmed competent species)*	0	1-2	≥3
Pig density (pigs per 100 people)	<10	10-29	≥30
Introduction risk (proximity and flyway connectivity)	>1,000km from an endemic country, outside East Asian-Australasian Flyway (EAAF) and West Pacific Ocean Flyway (WPOF)	One criterion met, or within the WPOF	<1,000km from an endemic country, within EAAF

*Countries with 1–2 confirmed competent ardeid species but ≥11 total ardeid species were upgraded to high suitability.

### Exposure assessment: Ecological suitability for JEV transmission

Exposure assessments in disease risk frameworks are typically human-focused; however, here we considered JEV exposure in non-human populations (vectors and vertebrate hosts) as a proxy for ecological transmission suitability, given that human cases arise as spillover from established enzootic or epizootic transmission cycles involving mosquitoes, waterbirds, and pigs. We therefore evaluated the potential for how JEV, once introduced, could be transmitted by different mosquito vector and vertebrate host populations.

Mosquito species data for each country and territory were obtained from *A guide to mosquitoes in the Pacific* [30]. Species were categorised as either competent vectors (i.e., those with demonstrated transmission competence in laboratory studies) or probable vectors (i.e., species from which JEV has been isolated in endemic Asian settings but lack experimental transmission data) [31]. Experimental transmission success rates for each mosquito species were extracted from a published review of JEV vector competence studies [31], which reported the proportion of mosquitoes with JEV detected in saliva following experimental infection, and a proxy for transmission success. For each study, we extracted the reported minimum and maximum transmission success percentages and calculated the midpoint value (mean of minimum and maximum) to represent that study’s transmission success rate. We then calculated species-level mean transmission success across all studies for each mosquito species. Vector suitability was categorised based on the number of JEV competent or potentially competent mosquito species per country, scaled as low (<5), moderate (5–9) or high (≥10) (Table 1).

Host data focused on ardeid (herons and egrets) species and pig populations. For the ardeids, species checklists for each country were obtained from AviBase [32], which list all known ardeid species for each PIC. Classifying a species as a competent JEV reservoir host was based on results in experimental infection studies and published reviews [19,23,33,34]. Ardeid host scores were determined in two steps: first, PICs were classified according to the richness of ardeid species with experimental evidence of JEV competence (only five known species: *Ardea alba* Great egret, *Ardea intermedia* Intermediate egret, *Nycticorax caledonicus* Nankeen night heron, *Bubulcus ibis* Cattle egret, *Ardea plumifera* Plumed egret), scored as low (0 confirmed host species), moderate (1–2 confirmed species), or high (≥3 confirmed species). Second, overall ardeid richness (including species with unknown competence) was used to refine this classification. PICs with moderate preliminary scores (1–2 confirmed hosts) but high total ardeid richness (≥11 species) were upgraded to high suitability, reflecting the potential for additional unconfirmed species to serve as reservoir hosts (Table 1). Countries with low total ardeid richness (<5 species) maintained their preliminary classification, as limited overall diversity constrains reservoir capacity regardless of confirmation status (Table 1). This two-step approach balances empirical evidence of host competence with the ecological reality that reservoir capacity depends on both proven and potential host diversity.

For pigs, we considered pig density to be most important for the amplification of JEV. Pigs per 100 people was calculated using 2023 pig stock values from the Food and Agriculture Organisation Corporate Statistical Database [35], divided by World Bank population estimates for 2023 per country [36], multiplied by 100. Where Food and Agriculture Organization Statistical Database (FAOSTAT database) data were unavailable (Guam, Northern Mariana Islands, American Samoa, Palau, and Wallis and Futuna), pig stock data were obtained from the most recent available national agricultural census or country profile reports: United States Department of Agriculture (USDA) National Agricultural Statistics Service Census for US territories (Guam, Northern Mariana Islands [37,38]), Food and Agriculture Organization country profile documents (American Samoa [39]), national population and housing censuses (Palau [40]), and Secretariat of the Pacific Community statistical databases (Wallis and Futuna [41]). Data were unavailable for Marshall Islands and Pitcairn Islands. Pig suitability was classified as low (<10 pigs per 100 people), moderate (10–29 pigs per 100 people), or high (≥30 pigs per 100 people). Table 1 summarises the classification thresholds applied to each risk component.

### Risk characterisation: Integration of release and exposure components

The release (introduction) and exposure (vector, host suitability) scores were combined to assign overall JEV transmission risk categories for each PIC. This risk characterisation distinguished between settings with different transmission profiles. PICs with moderate or high scores for vector diversity and ardeid host diversity, were classified as having *Endemic Potential*, conducive with ecological conditions that could support sustained wildlife-mosquito transmission cycles capable of long-term viral persistence. Countries with moderate or high pig populations and moderate or high vector diversity but low waterbird host diversity were classified as having *Epidemic Potential*, indicating capacity for pig-amplified outbreaks but insufficient wildlife reservoir diversity for long-term endemic maintenance. This category also included countries with extreme pig densities (>100 pigs per 100 people), but limited vector diversity where epidemic amplification could still be possible. Countries with low vector diversity, low waterbird diversity, and low or moderate pig density were classified as *Low Risk*, reflecting insufficient host-vector combinations to support sustained or epidemic transmission cycles. Countries and territories within each transmission profile were then stratified by release component scores (high, moderate or low) to distinguish between settings where both suitable ecology and probable introduction pathways converge from settings where geographic isolation or ecological constraints limit overall risk despite the presence of one favourable component.

## Results

This assessment classified the 22 PICs into three risk profiles for JEV: Endemic Potential, Epidemic Potential, and Low Risk. These profiles reflect the different combinations of vectors, hosts, and introduction pathways, and so imply different surveillance priorities. The sections below build toward this classification, beginning with the region’s documented JEV history before assessing each component of risk in turn.

### Baseline JEV status

JEV is endemic in PNG where serological, entomological and clinical evidence has accumulated since the late 1980s [41–43]. Historic evidence suggests that JEV likely reached PNG following eastward expansion through the Indonesia archipelago. This is supported by isolations of JEV in mosquitoes from Lombok, Indonesia in 1978 and then on Flores, Indonesia in 1981 [44]. Serological evidence of JEV infections in humans in PNG began to emerge by 1989 and then over the following decade extensive surveys revealed widespread and increasing seropositivity in populations across multiple regions of PNG [13]. In the Daru area of southern Western Province-located in the far southwest of PNG near the Torres Strait-22% of human sera collected in 1989 were positive for JEV antibodies. Further inland, in the Upper Fly River region of the same province, seroprevalence appeared to be increasing over time, with 8% of sera testing positive in 1990–1991 and 28% in 1997 [13,45]. Additional surveys found 17% seropositivity in Gulf Province (central southern PNG) in 1993 and 4% in Southern Highlands Province (central PNG) in 1994, while no antibodies were detected in sera collected from individuals living in northern or eastern PNG [13]. These findings suggest that JEV was initially established in the southwest of the country and gradually expanded its range.

Clinical cases of JEV have also been reported in PNG, providing further confirmation of active transmission. The first clinically confirmed cases occurred in late 1997 and early 1998 in Western Province, where four individuals were diagnosed with JEV, two of whom died [42]. Around the same time, unconfirmed cases of encephalitis were reported from Milne Bay Province (on the eastern tip of PNG), and a serological survey conducted during the suspected outbreak found that nearly 40% of sera tested positive for JEV antibodies [43]. In 2004, a case of JEV was diagnosed in a long-term resident living near Port Moresby, the capital city situated on the southern coast midway between Western and Milne Bay Provinces [46]. The patient had lived in the area for decades, had no recent travel history, and resided near a piggery with poor mosquito screening which are conditions conducive to local transmission. Although isolated, this case highlighted the potential for JEV to persist and emerge outside previously recognised transmission areas [46]. Two paediatric cases of JE have also been reported in children who presented to Port Moresby General Hospital with febrile encephalopathy between June 2007 and July 2008 [47]. Entomological surveillance has provided direct evidence of JEV circulation in PNG mosquito populations. Between 1996 and 1998, nearly 400,000 adult mosquitoes were collected from 26 sites in Western Province. Three JEV isolates were obtained from multiple species, including *Cx. annulirostris* and *Cx. sitiens* group mosquitoes [48]. While this early evidence of JEV circulation in PNG was predominantly concentrated in the southern and western regions of the country, more recent surveillance efforts have revealed a considerably broader geographic distribution of the virus.

Between 2019 and 2021, a pilot One Health surveillance project led by CSIRO’s Australian Centre for Disease Preparedness in collaboration with PNG’s National Agriculture Quarantine and Inspection Authority and the PNG Institute of Medical Research conducted sentinel animal and mosquito surveillance across multiple provinces [49]. Serological evidence of JEV infection was detected in sentinel pigs in National Capital District, Central, and Morobe provinces during 2019–2020, as well as in archived pig samples from Sandaun, New Britain, Enga, and Western Province collected between 2016–2020. Importantly, JEV RNA was detected in two pools of *Cx. gelidus* mosquitoes collected from Morobe Province in 2020, representing the first molecular detection of JEV in PNG mosquitoes since the late 1990s. These findings indicated that JEV transmission has not only been ongoing but is geographically more widespread than previously documented, extending from the southern coastal regions along the northern coast and potentially into parts of the highlands.

Beyond PNG, two documented outbreaks of JEV have occurred in the Pacific region: in Guam during 1947–1948, and in Saipan, part of the Northern Mariana Islands, in 1990. On Guam, the outbreak coincided with a mumps epidemic, and the JEV aetiology was only recognised after a fatal encephalitis case without mumps symptoms prompted virological investigation [50]. A total of 54 encephalitis cases were recorded among residents and non-residents, with 16 attributed to JEV and five deaths; virus was isolated from brain tissue of four fatal cases, confirming the virus as the causative agent. Follow-up studies found widespread JEV-neutralising antibodies in humans, pigs, and horses, and later entomological investigations identified *Cx. annulirostris* as the most likely mosquito vector on epidemiological grounds, although no virus was isolated from field-caught mosquitoes. No further JEV cases were reported, and children born after 1947 lacked JEV antibodies despite continued vector presence. The authors concluded that this represented a single, non-sustained epidemic following recent introduction of JEV into a previously naive island population, probably facilitated by wartime and postwar movement of people, animals, and mosquitoes from endemic parts of Asia [50].

For Saipan, 10 encephalitis cases occurred over a three-week period in October 1990, including two deaths [51]. Although delays and freeze-thaw damage to specimens prevented virus isolation, three cases met the confirmed case definition based on a fourfold rise in JEV neutralising antibodies without corresponding rises to dengue or Murray Valley encephalitis viruses, and seven additional cases were classified as probable on the basis of IgM and complement fixation results. A 1991 serosurvey found 96% of pigs and 4.3% of lifelong residents had JEV antibodies [51]. In contrast, none of 288 stored serum samples from 1984 were seropositive, and no further human or pig infections were detected in 1991, supporting the conclusion that JEV was recently introduced and that transmission ceased after rapid saturation of amplifying hosts. Entomological investigations documented abundant *Cx. tritaeniorhynchus* and identified this species as the probable vector, consistent with its role as the principal JEV vector in much of rural Asia. The authors considered several possible introduction routes, including viremic migratory birds, inadvertent importation of infected mosquitoes or eggs via air or sea transport, and argued that the epidemiologic and serologic patterns were most consistent with a rare introduction event into a previously naive island rather than long-term undetected circulation [51]. Historical serosurveys in Fiji in 1959–1960 also detected hemagglutination-inhibiting antibodies to JEV in 40% of human samples, suggesting prior exposure or cross-reactivity, although no virus was isolated from mosquitoes, and the presence of active transmission could not be confirmed [52].

To date, no confirmed cases or viral isolations of JEV have been reported from PICs beyond PNG, Guam and Saipan. However, the lack of confirmed detections does not necessarily indicate that transmission has never occurred, but rather it is possible that it has not been or is not currently investigated. Encephalitic illness in the region may go unrecognised or misdiagnosed due to low index of clinical suspicion, under-reporting, limited laboratory diagnostic capacity, and the overlap of clinical symptoms and signs for mosquito-borne diseases. Serological confirmation is particularly challenging in flavivirus-endemic areas because antibodies generated in response to one flavivirus (such as dengue) can cross-react with others, including JEV, especially in ELISA and hemagglutination inhibition assays [53]. These limitations complicate interpretation of serosurveys and highlight the need for improved surveillance and risk assessments across the region.

### Release assessment: Introduction pathways and potential

**Fig 1:**
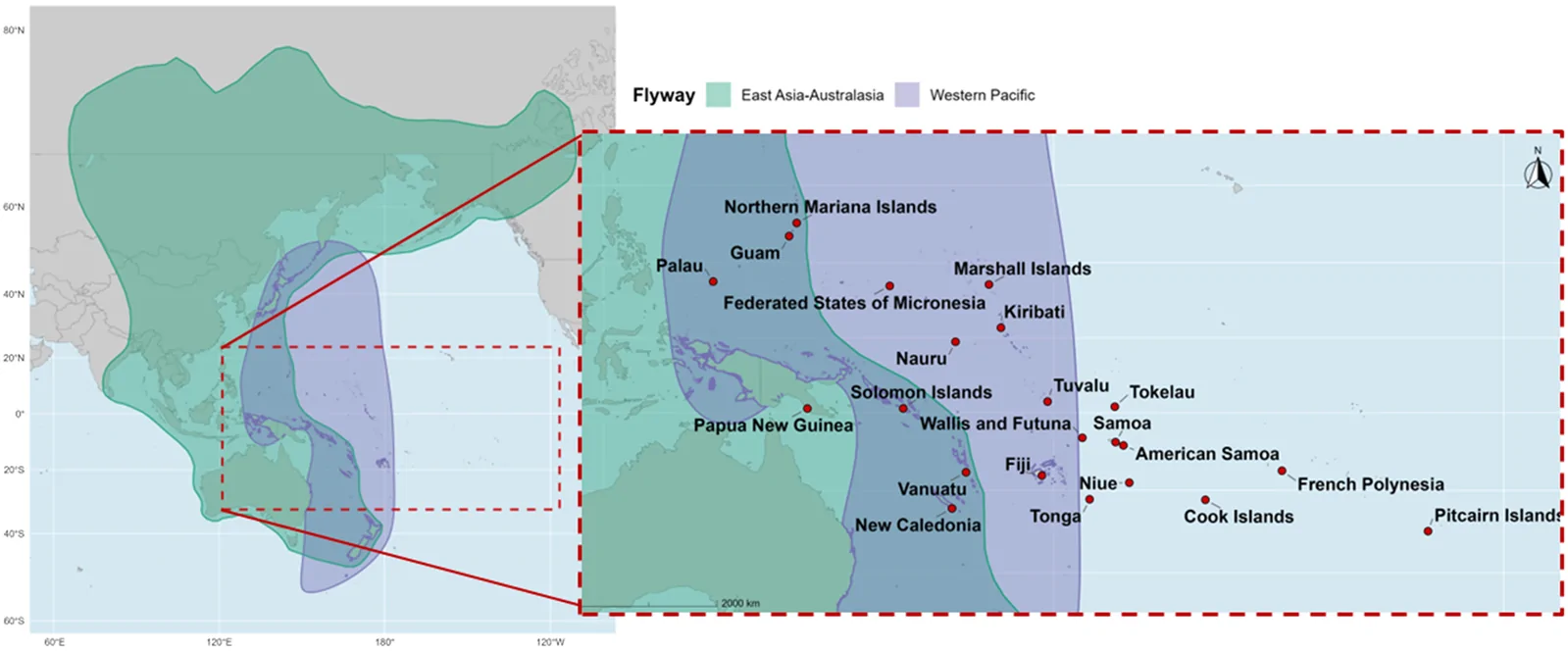
Estimated boundaries of the East Asia-Australasia and Western Pacific Ocean Flyways [29], and the location of each PIC within these (red bordered pop-out box). Basemap: Natural Earth (public domain; https://www.naturalearthdata.com; terms of use: https://www.naturalearthdata.com/about/terms-of-use/), accessed via the rnaturalearth package (v1.2.0) [55] in R (v4.6.0) [56]. Flyway boundaries reproduced from BirdLife International’s global flyways dataset, used with permission [57].

The long-distance movement of JEV via infected migratory waterbirds is widely recognised as a primary mechanism for viral introduction into new regions [13,14,17]. The connectivity of PICs to JEV-endemic regions via migratory pathways varies substantially across the region. Two of nine major flyways overlap with the Pacific: the East Asian-Australasian Flyway (EAAF), which holds over 50 million migratory waterbirds [54], and the West Pacific Ocean Flyway (WPOF) (Fig 1). While flyway designations indicate broad zones where species migrate, they do not capture the distance, specific routes, or seasonal patterns of individual species or populations, and rather, represent the collective movement of diverse bird communities [54], most of which are unknown JEV hosts [14].

The EAAF extends from the far east of Russia and Alaska southwards through East Asia and Southeast Asia to Australia and New Zealand, overlapping with multiple JEV-endemic countries including China, Japan, Korea, the Philippines, Indonesia, and mainland Southeast Asia, creating direct pathways for potential viral introduction via infected waterbirds (Fig 1) [58]. PICs with established EAAF connectivity include PNG, the Solomon Islands, New Caledonia, Vanuatu, Palau, Guam and Northern Mariana Islands, all of which lie along migration routes that overlap with JEV-endemic Asia [59]. These PICs face a higher risk of waterbird-mediated JEV introduction due to established migratory connectivity with JEV endemic areas. PICs overlapping with the WPOF, which extends from New Zealand and eastern Australia northward through the central Pacific Ocean to eastern Asia and Alaska [60], have a risk of JEV introduction from migratory birds. However, this flyway mostly supports shorebirds which are have not been experimentally tested for their JEV host competence, and only one documented ardeid species [29]. The WPOF is also characterised by extensive oceanic barriers, with vast stretches of open water separating widely dispersed island chains across the Pacific [58]. PICs situated in the WPOF include Fiji, Nauru, Tuvalu, Federated States of Micronesia, Kiribati and Marshall Islands [29]. The absence of regular JEV-relevant waterbird migratory pathways connecting these regions to endemic parts of Asia substantially reduces their virus introduction risk via this pathway. Nine PICs are outside of all migratory flyways these include Tonga, Samoa, Cook Islands, American Samoa, Wallis and Futuna, French Polynesia, Niue, Pitcairn Island and Tokelau (Table 2).

**Table 2:** Release risk assessment for JEV introductory pathways including migratory flyways and distance to JEV endemic countries.

Pacific Island country and territory	Migratory Flyway	Distance to JEV endemic country (km)	Release Risk Assessment*
New Caledonia	East Asian-Australasian Flyway (EAAF)	969	High
Northern Mariana Islands	EAAF	538	High
Palau	EAAF	655	High
Papua New Guinea	EAAF	0	High
Solomon Islands	EAAF	5	High
Guam	EAAF	1223	Moderate
Vanuatu	EAAF	1216	Moderate
Federated States of Micronesia	West Pacific Ocean Flyway (WPOF)	1183	Moderate
Fiji	WPOF	2116	Moderate
Kiribati	WPOF	1190	Moderate
Marshall Islands	WPOF	1440	Moderate
Nauru	WPOF	940	Moderate
Tuvalu	WPOF	1841	Moderate
American Samoa		4953	Low
Cook Islands		5564	Low
French Polynesia		6895	Low
Niue		4891	Low
Pitcairn Islands		8076	Low
Samoa		5057	Low
Tokelau		3126	Low
Tonga		4156	Low
Wallis and Futuna		2671	Low

* High Release Risk Assessment: within EAAF and <1,000km from a JEV endemic country; Moderate Release Risk Assessment: within the WPOF or within EAAF and >1,000 km from a JEV endemic country; Low Release Risk Assessment: > 1,000km from an endemic country, outside EAAF and WPOF.

Windblown mosquitoes can be transported over water for considerable distances, with Ritchie and Rochester [17] generating simulations of windborne mosquito dispersal of 200–300 km from PNG to Australia during the 1995 and 1998 JEV outbreaks. Windborne migration is a common phenomenon among diverse insect taxa, enabling passive aerial transport via seasonal atmospheric circulation patterns over hundreds to thousands of kilometres [61]. In the western Pacific, these wind circulation patterns create potential corridors for long-distance mosquito dispersal. From June to October, the southwest monsoon generates prevailing south-westerly winds across the region, with the monsoon trough extending eastward to near Guam and bringing gale-strength winds and monsoon surges that affect the western shores of Micronesian islands [62]. From November to March, northeast trade winds dominate, creating northeasterly flows from continental East Asia across the region. Passive windborne dispersal has been documented in mosquitoes, with *Ae. vigilax* capable of dispersing up to 50 km offshore [63]. The likelihood of JEV introduction via windborne mosquito dispersal depends heavily on geographic proximity to endemic regions and prevailing wind patterns. Five PICs are within 1,000 km of a JEV endemic country (Solomon Islands, New Caledonia, Palau, Vanuatu and Nauru; Table 2). The remaining 16 PICs are further than 1,000 km from a JEV endemic country with the furthest distance being >8,000km (Pitcairn Islands), where windborne mosquito introduction is highly improbable (Table 2).

While the introduction of JEV via infected mosquitoes in commercial aircraft or shipping containers remains possible, particularly for areas with direct transport links to JEV-endemic regions, we did not consider this in our release assessment. Further, the movement of infected livestock, particularly pigs, from endemic to non-endemic regions could theoretically introduce the virus. However, international animal health regulations and quarantine procedures may prevent such movements, and there is no evidence of JEV introduction via livestock trade in the Pacific context. Humans, while susceptible to infection, develop viremia levels that are both low in titre and short in duration (typically a few days), rendering them incapable of infecting mosquitoes and thus classified as dead-end hosts for transmission [64]. Consequently, human travel and migration are not considered to contribute meaningfully to geographic spread of JEV.

### Exposure assessment: Vector species presence and competence

**Fig 2:**
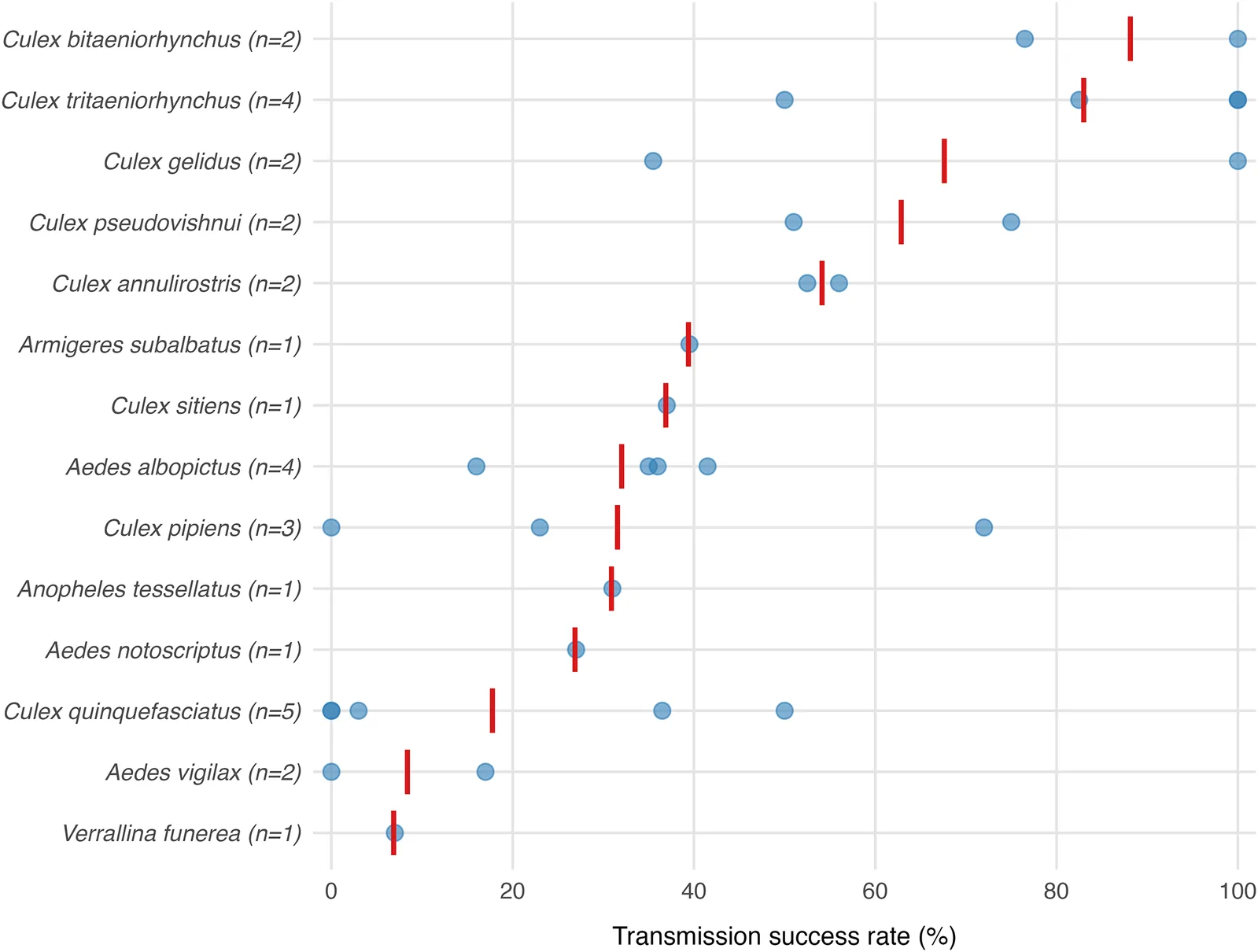
Transmission success rates for JEV-competent mosquito species present in Pacific Island countries and territories, based on experimental studies reviewed in Van den Eynde et al. (2022). Each blue circle represents the mean transmission rate from a single published experiment. Red vertical lines represent the mean across all experiments for each species. Species are ordered by mean transmission success (highest to lowest).

JEV vector potential varies widely across the region, shaped by differences in mosquito species composition and their vector competence for JEV, which is poorly understood. At least 21 mosquito species present in the region have been identified as confirmed or potential JEV vectors, based on a combination of laboratory transmission experiments and field detections (Tables 3, S1, S2). Of these, *Cx. tritaeniorhynchus* is considered the most important JEV vector because it consistently demonstrates high infection and transmission rates in laboratory studies, often exceeding transmission rates of 80%, and is considered a well-established JEV vector in endemic regions of Asia (Fig 2; [31]). Interestingly, the only three PICs where *Cx. tritaeniorhynchus* has been reported in (PNG, the Northern Mariana Islands, and Guam) are also the only ones in which human outbreaks of JEV have been documented; however, the true distribution and abundance of *Cx. tritaeniorhynchus* within these settings remain uncertain, and recent surveys suggest the species is rarely or infrequently detected in PNG [65]. Other species such as *Cx. gelidus* and *Cx. bitaeniorhynchus* have also demonstrated very high transmission potential in laboratory settings, with some studies reporting transmission rates of up to 100% and 89%, respectively (Fig 2; [31]). *Cx. gelidus* is present in PNG and New Caledonia, but has so far only been implicated in JEV transmission in northern Australia and PNG, where it has been associated with virus detections and demonstrated competence in experimental studies [48,49,66]. *Cx. bitaeniorhynchus* has only been identified in PNG, New Caledonia, and Palau. *Cx. annulirostris*, the primary vector of JEV in Australia, is among the most widespread JEV vector in the Pacific. It is present in 18 of the 22 PICs, notably absent from the Marshall Islands, Niue, Pitcairn Islands, and Tokelau. Although laboratory transmission rates for *Cx. annulirostris* vary, ranging from 24% to over 80% depending on the study, its broad distribution, ecological flexibility, and association with historical outbreaks make it a key species of concern throughout the region.

**Table 3:** Presence of mosquito species with confirmed or potential JEV vector competence across Pacific Island countries and territories. Orange-coloured cells indicate species with demonstrated transmission competence in laboratory studies. Blue cells denote species for which JEV has been detected in field-collected mosquitoes, but without experimental confirmation of transmission. The rightmost column shows the total number of JEV-competent or potentially competent mosquito species recorded in each country.

	*Aedes*					*Anopheles*				*Culex*								*Mansonia*			Other			Total
	*Ae. albopictus*	*Ae. kochi*	*Ae. notoscriptus*	*Ae. vexans*	*Ae. vigilax*	*An. barbirostris*	*An. subpictus*	*An. tessellatus*	*An. vagus*	*Cx. annulirostris*	*Cx. bitaeniorhynchus*	*Cx. gelidus*	*Cx. quinquefasciatus*	*Cx.sinensis*	*Cx. sitiens*	*Cx. tritaeniorhynchus*	*Cx. whitmorei*	*Ma. annulifera*	*Ma. indiana*	*Ma. uniformis*	*Verrallina funerea*	*Armigeres subalbatus*	*Coquillettidia ochracea*	
American Samoa																								4
Cook Islands																								3
Federated States of Micronesia																								4
Fiji																								6
French Polynesia																								3
Guam																								11
Kiribati																								4
Marshall Islands																								3
Nauru																								4
New Caledonia																								8
Niue																								2
Northern Mariana Islands																								5
Palau																								6
Papua New Guinea																								19
Pitcairn Islands																								1
Samoa																								5
Solomon Islands																								7
Tokelau																								1
Tonga																								5
Tuvalu																								5
Vanuatu																								6
Wallis and Futuna																								4

Across PICs, *Aedes* mosquitoes are widespread but generally show lower experimental competence for JEV than key *Culex* vectors, with most *Aedes* species exhibiting modest or highly variable transmission success in laboratory studies (Fig 2) [12,67]. *Aedes vexans* is the most frequently recorded *Aedes* species in the region (Table 3), occurring in many jurisdictions in Melanesia, Micronesia, and Polynesia. The species has been implicated as a potential vector after JEV was isolated from field-caught *Ae. vexans* in Taiwan [68] and early experimental work in Guam demonstrated JEV transmission to infant mice [69], although experiments with North American populations failed to confirm consistent infection or transmission [70]. *Aedes albopictus*, which is present in half of the PICs (Table 3), can become infected with JEV and occasionally transmit virus in experimental settings, but reported transmission rates are typically lower than major *Culex* vectors, and there is little field evidence linking it to human outbreaks [12,71]. In Australia, *Ae. vigilax* and *Ae. notoscriptus* have been discussed as possible bridge vectors because of their high abundance, single field detection in *Ae. vigilax*, and demonstrated experimental competence, yet they are still regarded as minor contributors to JEV transmission [12]. More broadly, experimental work has shown that some *Aedes* mosquitoes can transmit JEV vertically to their progeny, suggesting that desiccation-resistant eggs may help maintain the virus in the environment even when horizontal transmission is low, although the epidemiological importance of this mechanism is not yet well quantified [72].

The calculated risk posed by JEV vectors in the Pacific is dependent on the diversity of proven and probable JEV-competent vector species in each PIC. High vector suitability (≥10 competent species) was identified in PNG and Guam. Nine PICs were considered to have moderate vector suitability (5–9 JEV vector species; Nauru, Northern Mariana Islands, Samoa, Tonga, Tuvalu, Fiji, Palau, Vanuatu, Solomon Islands and New Caledonia). Low vector suitability was assigned to the remaining ten PICs (Niue, Pitcairn Islands, Tokelau, Cook Islands, French Polynesia, Marshall Islands, American Samoa, Federated States of Micronesia, Kiribati and Wallis and Futuna), six of which are reported to have only 1–3 potential vector species. Tokelau and the Pitcairn Islands each have only a single JEV-competent species reported, which has either been linked to JEV outbreaks in other regions or is experimentally competent. *Aedes vexans*, found in Tokelau, has demonstrated the ability to transmit JEV under experimental conditions and has been detected in field-collected mosquitoes during outbreaks in Taiwan [68,73]. Likewise, *Cx. quinquefasciatus*, reported in the Pitcairn Islands, has been associated with JEV activity through field isolations in India, Thailand, and Vietnam [73]. These patterns suggest that while higher vector diversity may increase the range of mosquito-host interactions that could support viral persistence or spillover within a jurisdiction, areas with only one or a few vector species can still be at meaningful risk if those species are competent.

While the presence of JEV-competent mosquitoes across the region helps estimate the exposure risk, understanding the ecological traits of these species is also crucial for interpreting where and how transmission may occur. Several species, such as *Cx. annulirostris*, *Cx. tritaeniorhynchus*, and *Cx. bitaeniorhynchus*, are typically associated with freshwater habitats like rice paddies, swamps, and floodplains [73]. These environments are common in rural or agricultural areas, where pigs and ardeid birds serve as key amplifying and reservoir hosts for JEV. By contrast, species such as *Cx. quinquefasciatus* are urban-adapted mosquitoes that breed in polluted water sources including drains, septic tanks, and household containers, and feed on both birds and humans, making them potential bridge vectors in densely populated settings [12]. Salt-tolerant species like *Ae. vigilax* and *Ae. vexans* inhabit coastal or brackish environments and are capable of long-distance dispersal, allowing them to reach areas distant from breeding sites [74]. These distinctions highlight that vector species presence alone does not determine transmission risk. The interplay between local landscape features, land use, climate factors, vector abundance, host availability, and mosquito behaviour shapes the conditions under which transmission may emerge or persist. Tailored vector surveillance and control strategies that account for these ecological differences are essential to effectively mitigate JEV risk across diverse regional settings.

### Exposure assessment: Vertebrate host populations

The presence and diversity of waterbird host species varied across PICs. Of the species with known experimental competence for JEV, the most common was the Great egret, reported in 10 of the 22 PICs (S3 Table), which is also a species that has demonstrated some of the highest and longest lasting reported viraemia under experimental conditions [23]. The Nankeen night heron was the second most reported species with known JEV competence, reported in 7 of the 22 PICs. The most common ardeid species, with no experimental studies evaluating competence was *Egretta sacra*, the Pacific Reef heron, reported in all 22 PICs.

**Fig 3:**
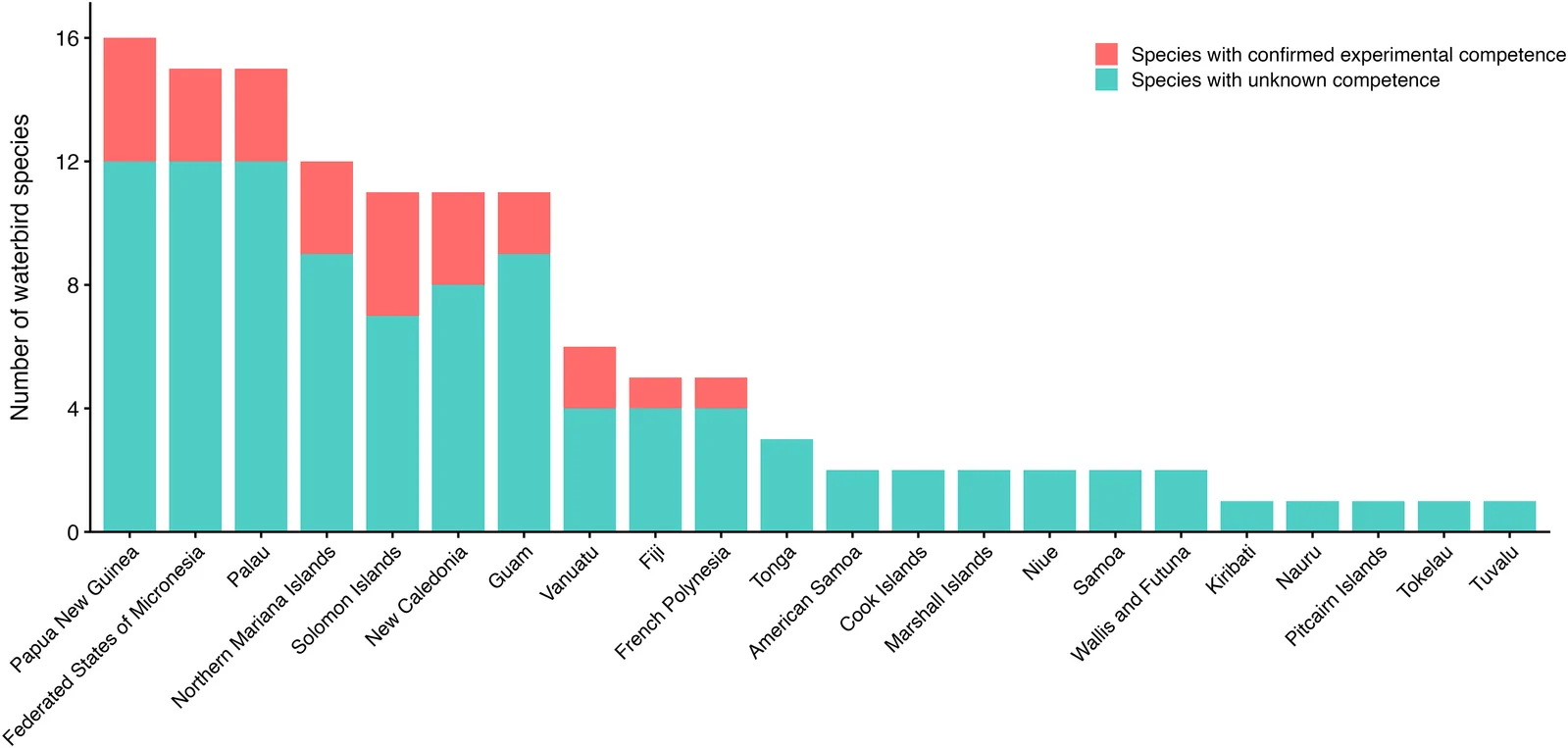
Ardeid waterbird species richness in Pacific Island countries and territories. Red bars indicate species with confirmed experimental JEV competence based on reviewed evidence from [23]; teal bars indicate species with unknown competence. Countries ordered by total richness, then by number of confirmed competent species (both descending).

Five PICs were considered to have high reservoir suitability: PNG (4 confirmed JEV competent host species and 16 total ardeid species), Palau and Federated States of Micronesia (both with 3 confirmed host species, 15 total ardeid species), Northern Mariana Islands (3 confirmed JEV competent host species, 12 total ardeid species) and Solomon Islands (4 confirmed JEV competent host species and 10 total ardeid species; Fig 3). Another five PICs were considered to have moderate bird reservoir suitability (New Caledonia, Guam, Vanuatu, Fiji, and French Polynesia) because at least 1 confirmed JEV host species was reported in the species lists, along with moderate overall ardeid species diversity (5–10 species). Low reservoir suitability was assigned to twelve PICs with no confirmed competent hosts and low total ardeid species richness (<3 species): Tonga, American Samoa, Cook Islands, Marshall Islands, Niue, Samoa, Wallis and Futuna, Kiribati, Nauru, Pitcairn Islands, Tokelau, and Tuvalu.

**Fig 4:**
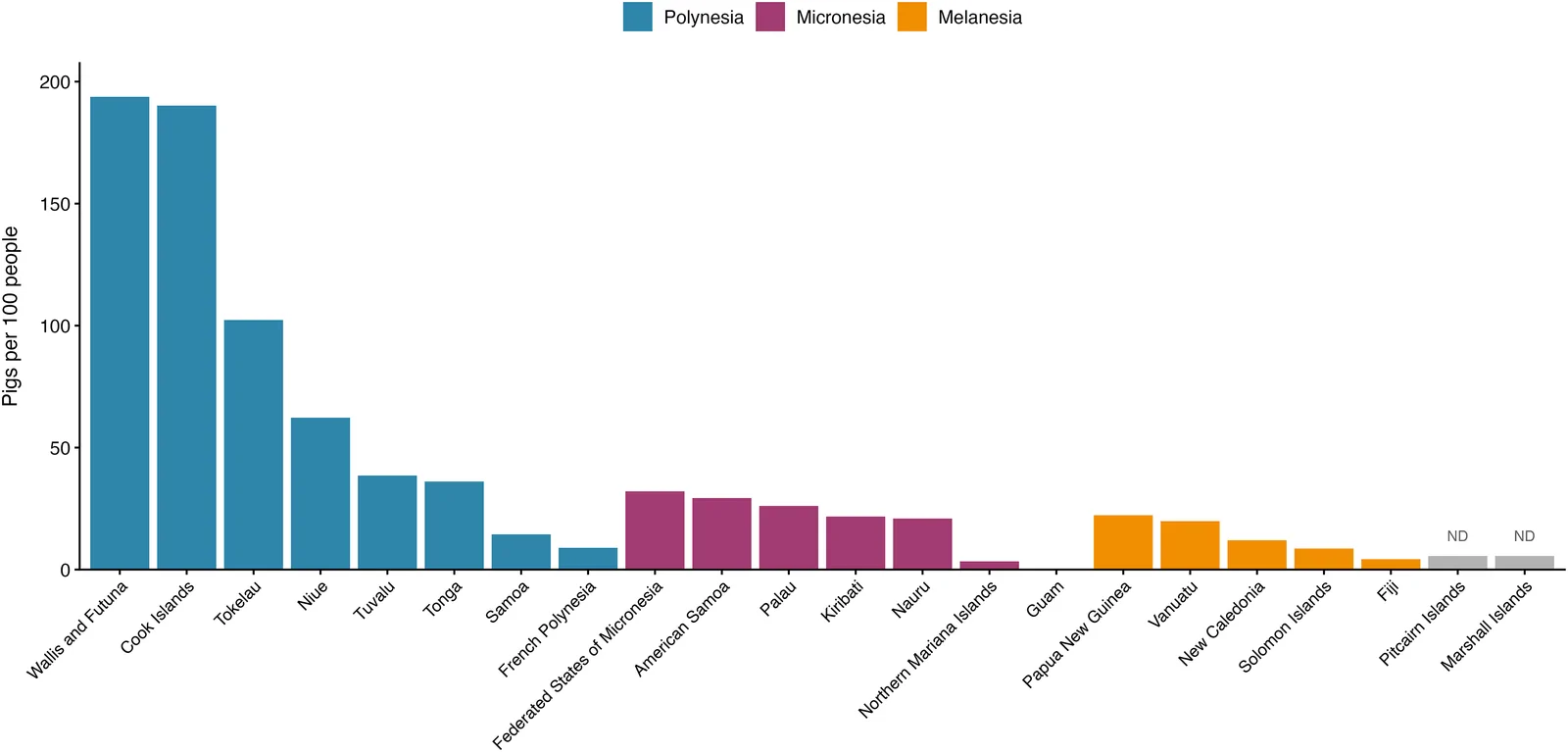
Pig density across Pacific Island countries and territories, grouped by geographic subregion (Polynesia, Micronesia, Melanesia) and ordered by pig density within each region. Pig density is expressed as pigs per 100 people based on 2020 data from FAOSTAT (n = 15) or national agricultural census reports (n = 5). No data were available for Marshall Islands and Pitcairn Islands.

Pig densities per population were available for 20 of the 22 PICs, with no pig production data available for the Marshall Islands or Pitcairn Islands (S4 Table). High pig densities (≥30 pigs per 100 people) were observed across several Polynesian countries and territories (Wallis and Futuna, Cook Islands, Tokelau, Niue, Tuvalu, Tonga), indicating substantial JEV amplification potential in these settings despite limited waterbird diversity (Fig 4). Moderate pig densities (10–29 pigs per 100 people) were identified in Federated States of Micronesia, American Samoa, Palau, PNG, Kiribati, Nauru, and Vanuatu. Low pig densities (<10 pigs per 100 people) were identified in the remaining PICs, including Melanesian nations (Solomon Islands, Fiji, New Caledonia), Polynesian countries and territories (Samoa, French Polynesia), and Micronesian countries and territories (Northern Mariana Islands, Guam). These figures represent reported domestic pigs at a national scale and may not capture feral pig populations or some household pig ownership, which could contribute to viral maintenance in some regions.

### Risk characterisation: Integrated assessment of JEV transmission potential

Integration of release (introduction pathways) and exposure (vector and host assessments) identified distinct risk profiles across the Pacific region (Table 4). Eight PICs were classified as having *Endemic Potential* because they possess moderate-to-high vector suitability (≥5 competent mosquito species), combined with moderate-to-high ardeid host suitability (≥1 confirmed competent species or ≥5 total ardeid species). This profile indicates ecological conditions capable of supporting sustained JEV transmission cycles through wildlife-mosquito maintenance. Within this group, introduction risk stratification identified five PICs at higher introduction risk due to both EAAF connectivity and a proximity of <1,000km to endemic regions: PNG (already endemic with confirmed JEV circulation), Solomon Islands, New Caledonia, Palau and Northern Mariana Islands. Three PICs were classified as moderate introduction risk, meeting only one criterion: Fiji, Vanuatu and Guam (Table 4). These eight settings represent the highest priority for enhanced surveillance and preparedness planning, particularly the five at higher introduction risk where both suitable ecology and probable introduction pathways overlap.

**Table 4:** Integrated JEV risk assessment based on vector diversity, waterbird hosts (ardeids), pig density, climate suitability, and introduction risk. Components scored as Low (yellow), Moderate (orange), or High (red) (detailed scoring values in S5 Table). Introduction risk assessed based on proximity to endemic regions and flyway connectivity. Overall classifications: Endemic Potential – conditions support sustained wildlife-mosquito transmission cycles; Epidemic Potential – conditions support pig-amplified outbreaks but limited long-term maintenance due to low host diversity; Low Risk – limited host-vector combinations constrain transmission potential. *Wallis and Futuna and Cook Islands were classified as having Epidemic Potential due to very high pig densities (>190 pigs per 100 people) along with multiple vector species (four and three, respectively).

Pacific Island country or territory	Vector Suitability	Avian Host Suitability	Pig Host Suitability	Introduction Risk	Suitability Classification
**Papua New Guinea**	High	High	Moderate	High	**Endemic**
**New Caledonia**	Moderate	High	Moderate	High	**Endemic**
**Solomon Islands**	Moderate	High	Low	High	**Endemic**
**Palau**	Moderate	High	Low	High	**Endemic**
**Northern Mariana Islands**	Moderate	High	Low	High	**Endemic**
**Guam**	Moderate	High	Low	Moderate	**Endemic**
**Vanuatu**	Moderate	Moderate	Moderate	Moderate	**Endemic**
**Fiji**	Moderate	Moderate	Low	Moderate	**Endemic**
**Tonga**	Moderate	Low	High	Low	**Epidemic**
**Federated States of Micronesia**	Low	High	High	Moderate	**Epidemic**
**Tuvalu**	Moderate	Low	High	Low	**Epidemic**
**Samoa**	Moderate	Low	Moderate	Low	**Epidemic**
**Nauru**	Moderate	Low	Moderate	Moderate	**Epidemic**
**Cook Islands**	Low	Low	Very High*	Low	**Epidemic**
**Wallis and Futuna**	Low	Low	Very High*	Low	**Epidemic**
**American Samoa**	Low	Low	Moderate	Low	**Low Risk**
**Kiribati**	Low	Low	Moderate	Moderate	**Low Risk**
**French Polynesia**	Low	Moderate	Low	Low	**Low Risk**
**Marshall Islands**	Low	Low	Not available	Moderate	**Low Risk**
**Niue**	Low	Low	High	Low	**Low Risk**
**Pitcairn Islands**	Low	Low	Not available	Low	**Low Risk**
**Tokelau**	Low	Low	High	Low	**Low Risk**

Seven PICs were classified as having *Epidemic Potential* (Table 4), characterised by ecological conditions that could support pig-mosquito amplification and human spillover but lacking sufficient waterbird diversity for long-term endemic maintenance. This included four settings with high pig densities (≥30 pigs per 100 people) and low ardeid host suitability (<5 species; Wallis and Futuna, Cook Islands, Tuvalu, Tonga), of which two (Tuvalu and Tonga) had moderate vector diversity (5 species), and the other two (Wallis and Futuna, Cook Islands) had very high pig densities (>190 pigs per 100 people) and several vector species (four and three vector species, respectively). One setting, Federated States of Micronesia, qualified through high pig density (≥30 pigs per 100 people) and high waterbird diversity despite having low vector diversity (<5 potential species). Nauru and Samoa had both moderate pig densities and moderate vector suitability. Introduction risk within this group varied: three countries (Nauru, Tuvalu, and Federated States of Micronesia) were classified as moderate introduction risk due to being within the WPOF, while four were classified as low introduction risk due to distances from endemic regions and absence of EAAF or WPOF connectivity (Wallis and Futuna, Cook Islands, Tonga and Samoa). While these PICs could experience epidemic activity following introduction, geographic isolation reduces the probability of natural viral introduction via migratory waterbirds or windborne mosquitoes.

Seven PICs were classified as *Low Risk* for sustained JEV transmission (Table 4) due to low vector suitability (<5 competent species) and either low ardeid host suitability (0 confirmed competent species and <5 total ardeid species) or low pig density (<10 pigs per 100 people). All settings in this category also faced low-moderate introduction risk with five located >3,000 km from a JEV endemic country and lacking EAAF or WPOF connectivity. This combination of limited ecological suitability and geographic isolation substantially reduces the probability of JEV establishment in these settings: American Samoa, Kiribati, French Polynesia, Marshall Islands, Niue, Pitcairn Islands, and Tokelau.

## Discussion

The recent emergence of JEV genotype IV in temperate Australia demonstrated that JEV can establish outside its historical geographical range in Asia. This raises urgent questions about whether the Pacific region represents the next frontier for JEV expansion. Our qualitative risk assessment identified 15 of 22 PICs with ecological conditions that could sustain JEV transmission through either endemic or epidemic cycles following successful viral introduction. This is a substantial proportion and indicates widespread regional vulnerability. However, the nature of the vulnerability differs across settings. Eight PICs could support sustained endemic circulation through wildlife-mosquito transmission cycles, seven could experience epidemic outbreaks supported by pig-mosquito amplification, and seven face ecological constraints that would limit even sporadic transmission. These distinctions are important because they determine appropriate public health responses, how resources should be allocated, and what surveillance strategies would be most effective for JEV in each setting given limited capacity and resources.

The three-tier risk classification (release, exposure and risk characterisation) applied here has direct operational implications for Pacific Island health systems and regional surveillance systems such as the Pacific Public Health Surveillance Network. These classifications indicate that some locations face higher relative risk than others based on ecological suitability, rather than predicting absolute probability of JEV establishment. Given than JEV has not been reported in the great majority of PICs, absolute risk levels remain uncertain across the region. Papua New Guinea represents the only PIC with confirmed endemic circulation, having had documented cases and detections since the late 1980s [45,46,49]. The qualitative risk assessment approach applied here successfully identified PNG as having endemic potential, providing empirical validation of the framework’s ability to distinguish transmission scenarios based on ecological factors. This concordance between predicted risk category and observed epidemiological patterns supports the utility of the classification system for evaluating transmission potential across other Pacific territories where JEV status remains unknown.

What distinguishes *Endemic Potential* settings is not simply the presence of competent mosquitoes or suitable hosts, but the high diversity of both. Multiple vector mosquito species exploiting different habitats combined with substantial ardeid species diversity create conditions where eradication of JEV would be highly challenging if established. Human vaccination reduces clinical disease but cannot eliminate circulation in wildlife populations. If JEV becomes established in the PICs characterised as having *Endemic Potential* (New Caledonia, Solomon Islands, Palau, Northern Mariana Islands, Guam, Vanuatu and Fiji), these settings would require sustained surveillance infrastructure, ongoing diagnostic capacity, and long-term public health strategies rather than time-limited outbreak responses. Integrated One Health surveillance approaches combining wildlife, mosquito, sentinel animal, and human monitoring may represent an ideal model for early detection in these settings. In PNG, One Health surveillance programs have successfully detected JEV circulation across multiple provinces through coordinated entomological and animal serological monitoring [49], though such comprehensive systems remain resource-intensive and may not be immediately feasible for all settings with limited surveillance capacity. A more feasible approach in *Endemic Potential* PICs could be targeted vector control and environmental management strategies that focus on high-risk interface zones, particularly wetlands and agricultural areas where mosquito breeding habitats, ardeid waterbird roosting sites, and pig farming operations converge, as these ecological hotspots present the greatest risk for establishing and amplifying transmission cycles that could subsequently spill over into human populations.

The seven *Epidemic Potential* PICs identified (Tonga, Federated States of Micronesia, Tuvalu, Cook Islands, Wallis and Futuna, Samoa and Nauru) would be best positioned to focus on rapid outbreak detection and response capacity rather than sustained surveillance infrastructure. Surveillance approaches for these settings could include syndromic surveillance systems that capture human acute encephalitis syndrome presentations and reproductive disease presentations in breeding pigs through passive clinical public and animal health reporting networks, and ongoing vector surveillance. The significant human case fatality rates of 20–30% in those with symptomatic JEV [1] combined with reduced intensive care capacity in PICs mean that even small outbreaks could overwhelm health systems. Beyond surveillance, essential preparedness measures could include establishing rapid diagnostic protocols for arboviral encephalitis [75], and developing specimen collection and transport protocols to regional reference laboratories with JEV diagnostic capacity. These activities could be integrated within existing clinical training programs and regional networks, where feasible, and incorporate a One Health approach involving collaboration between public and animal health sectors to leverage available resources, data sharing and laboratory capacity. Opportunities for capacity building and advocacy regarding JEV surveillance and preparedness could also be sought through established regional networks including the Pacific Public Health Surveillance Network, PACMOSSI, and the Pacific Vector Network, which connects PICs to strengthen vector-borne disease prevention and control [76].

Vaccination strategies across PICs face substantial supply and deployment constraints that would limit reactive outbreak response options [77]. Effective JEV vaccines are available which demonstrate high efficacy (>80–95%) and durable protection [4]. However, none of the major vaccine producers maintain finished product stockpiles, production timelines require months, and countries must typically order in advance [4]. In the event of a significant outbreak, mechanisms exist to obtain emergency vaccine supplies, and dose-sparing strategies such as intradermal vaccination can extend limited supplies by requiring lower doses per person [78,79]. Given the resource constraints, regulatory requirements and competing health priorities facing PICs, pre-emptive JEV vaccination is unlikely to be considered in the absence of confirmed transmission. Regional preparedness planning that includes vaccine access pathways and early outbreak detection therefore remains the most feasible approach in these settings.

The limiting risk factors in the seven *Low Risk* PICs (American Samoa, Kiribati, French Polynesia, Marshall Islands, Niue, Pitcairn Islands and Tokelau) suggests that if JEV were introduced, establishment of sustained transmission cycles would face substantial ecological constraints. The majority of PICs in this category also face low introduction potential due to extreme distances from endemic regions and no migratory flyway connectivity, further reducing the probability of natural viral arrival. However, this *Low Risk* classification should not be interpreted as absolute protection, as sporadic human cases could theoretically occur if infected mosquitoes were transported via aircraft or shipping from endemic regions during transmission seasons, particularly to PICs with regular direct air traffic from endemic Asia [18]. The lower risk designation reflects reduced probability of both successful introduction and ecological capacity for establishment rather than impossibility of any JEV activity. Surveillance systems capable of detecting arboviral encephalitis through syndromic surveillance and regional laboratory networks remain valuable for these settings to ensure that sporadic introductions or unrecognised circulation would be detected.

### Uncertainties and limitations

Several important uncertainties and limitations constrain interpretation of this qualitative risk assessment and highlight areas for further investigation. Our exposure assessment relies on vector and host presence data without detailed information on population abundance, seasonal dynamics, or fine-scale spatial distribution within PICs. Mosquito vector distributions can change rapidly, and those data collated through the *PacMOSSI Guide to Mosquitoes in the Pacific* were based on historic mosquito surveys and may not reflect current vector distributions in all areas. Experimental vector competence studies, while providing essential evidence of transmission capacity under controlled conditions, often use laboratory-adapted mosquito colonies maintained for multiple generations that may exhibit different infection and transmission rates than wild populations. Environmental factors including temperature, humidity, and mosquito nutritional status can substantially influence vector competence but are not captured in literature-derived transmission success rates. Similarly, ardeid waterbird species checklists indicate potential reservoir presence but do not capture migratory timing, seasonal abundance fluctuations, stopover site usage, or actual infection prevalence in wild populations. Movement ecology studies using satellite tracking or GPS tags could provide more precise data on connectivity between Pacific Islands and JEV-endemic regions, refining introduction risk estimates beyond broad flyway designations.

Our release assessment evaluated introduction pathways qualitatively, considering both migratory waterbird movements (assessed via flyway connectivity and proximity to endemic regions) and windborne mosquito dispersal (assessed via geographic distance and prevailing wind patterns), but precise probability estimates for viral arrival via different mechanisms remain uncertain. Quantitative risk assessment approaches that model introduction probability as a function of migratory bird abundance, infection prevalence in source populations, flight distances, boat transportation, stopover duration, and susceptible host availability at destination sites would provide more rigorous estimates but require empirical data that are currently unavailable for most Pacific settings.

Most critically, limited diagnostic and surveillance capacity across PICs means that subclinical transmission or low-level circulation could already be occurring undetected. This uncertainty is particularly relevant for settings classified as *Endemic Potential*, such as Solomon Islands, where ecological conditions appear suitable but no systematic JEV surveillance has been conducted. Our assessment characterises ecological suitability for transmission rather than confirmed absence of JEV, and settings we classify as lower risk may nonetheless harbour unrecognised viral circulation. The high proportion of JEV infections that are asymptomatic (>99% of infections in endemic Asian settings) combined with limited capacity for arboviral diagnostics means that sporadic human cases could easily be misclassified as other febrile illnesses or neurological syndromes. Similarly, cases of reproductive disease in breeding sows caused by JEV infection may be attributed to other aetiologies or production issues. Serological surveys of humans, domestic animals, and wildlife would provide valuable evidence of prior exposure but face challenges from cross-reactivity with other flaviviruses, particularly dengue which is endemic across much of the Pacific region [53], and Murray Valley encephalitis, which has also been found in PNG [48,80,81]; and is closely-related to JEV. Country-level analyses also mask substantial subnational heterogeneity, particularly relevant for ecologically diverse nations like PNG where transmission risk varies dramatically between coastal lowland areas with extensive wetlands and high waterbird diversity, highland regions with different mosquito assemblages and cooler temperatures that may constrain viral replication, and offshore islands with distinct ecological profiles and limited connectivity to mainland transmission cycles. Similarly, within-country variation in pig density, agricultural practices, human population distribution, and healthcare access creates heterogeneous risk landscapes that national-level classifications cannot capture. Subnational risk assessments using higher resolution spatial data on vector distribution, host populations, and environmental suitability would provide more actionable information for targeting surveillance and interventions but require detailed ecological data that are currently unavailable for most countries. These blanket national estimates should be interpreted as general indicators of ecological suitability rather than precise predictions applicable uniformly across all regions within a country. Updated risk assessments incorporating emergent surveillance data would be needed if JEV was detected in any of the PICs and for those (namely, PNG) where JEV is already known to circulate.

### Future surveillance and research priorities

Several surveillance and research priorities emerge from this assessment that would refine understanding of JEV risk in Pacific settings and support evidence-based preparedness planning. First, one potential approach for surveillance would be to incorporate JEV antigens into existing multiplex serological surveillance platforms. This approach has been implemented across Pacific Island settings and could be expanded to include systemic JEV surveillance [82,83]. This would also require the addition of other circulating flavivirus antigens to address serological specificity or rely on confirmatory testing using specific assays at a reference laboratory. Second, field and experimental validation studies of vector and host competence in Pacific ecosystems are also critically needed, as most experimental transmission data derive from Asian populations that may differ in susceptibility due to genetic variation or prior exposure history. Collecting and testing wild-caught mosquitoes from Pacific islands for JEV susceptibility in laboratory trials, combined with bloodmeal analysis to characterise host-feeding patterns, would provide locally relevant vector competence data. Similarly, serological surveys of ardeid waterbirds, domestic pigs, and other potential vertebrate hosts in high-risk settings could detect evidence of prior JEV exposure that would indicate unrecognised circulation or past introductions, though interpretation would require careful differentiation from cross-reactive antibodies generated by other flaviviruses [53,84]. Experimental infection studies on other ardeid species which commonly occur in the region, such as the Pacific Reef heron which is reported on all 22 PICs would be critical to inform the potential transmission ecology. Third, quantitative modelling of introduction probabilities via different pathways could also provide more rigorous risk estimates to guide preparedness investments. Approaches could include mechanistic models of migratory waterbird movement calibrated with satellite tracking data, atmospheric dispersion models of windborne mosquito transport using historical meteorological data, and stochastic simulation models of aircraft-mediated introduction that incorporate flight schedules, mosquito survival parameters, and seasonal transmission patterns in endemic regions. Finally, research on the role of bats and other non-traditional hosts in JEV ecology is needed, as recent detections in Indonesian flying fox populations suggest these highly mobile mammals may contribute to viral dispersal or maintenance in ways not captured by current surveillance paradigms focused on poultry and pigs.

## Conclusion

Taken together, our findings show that JEV transmission potential in the Pacific is not uniform but instead shaped by the interplay of mosquito vectors, and waterbird and pig hosts, with 68% of the 22 PICs presenting conditions which can support JEV transmission. These insights build on the historical literature that long recognised Southeast Asia and parts of Oceania as high-risk zones, but they extend the discussion by highlighting the differing risks across the Pacific in the context of a shifting JEV distribution that now includes temperate Australia. Addressing JEV potential in the region requires strengthening human, wildlife and vector surveillance capacity, and proactive regional preparedness and cooperation around outbreak response actions, including vector and environmental management strategies and vaccine access.

## Supporting information

S1 Table Vector transmission success. Transmission rate reported for each species and experiment.
(XLSX)

S2 Table Vector species per country. Total number of confirmed (species with experimental demonstration of JEV transmission) and probable (species with field detection of JEV) mosquito vectors per PIC.
(XLSX)

S3 Table Ardeid species richness. Ardeid species reported in species checklists for each PIC and whether there is known JEV competence.
(XLSX)

S4 Table Pigs per 100 people in each PIC, based on World Bank population estimates 2020, and pig production statistics from FAO (2020), or agriculture report where FAO is unavailable. (XLSX)

S5 Table Summary of all risk assessment components per PIC, including vector, host and introductory pathway assessments and categorization. (XLSX)
