# Supplementary material for: Qualitative risk assessment of Japanese encephalitis virus introduction and transmission in Pacific Island countries and territories
Source: PLoS Negl Trop Dis. 2026 Sep 3;20(9):e0014162. doi: 10.1371/journal.pntd.0014162 (PMC13588533; doi:10.1371/journal.pntd.0014162)
Supplement: Supplementary file 1 [file pntd.0014162.s007.docx]

#### RE: PNTD-S-26-00682: Qualitative risk assessment of Japanese encephalitis virus introduction and transmission in Pacific Island Countries and areas

#### Response to reviewers

We thank the reviewers for their careful reading and constructive feedback, and are pleased that all three found the study to be clear, rigorous, and a valuable contribution. Their suggestions have improved the clarity and precision of the manuscript, and we have addressed each point in turn below. Changes made to the manuscript are visible in the accompanying tracked-changes version.

Line numbers are in reference to the clean version of the resubmitted manuscript.

#### Reviewer 1

**Editorial and data presentation modifications**

*Comment 1: Terminology consistency.* We have standardised all instances of "Pacific Island Countries and areas" throughout the title, abstract, and main text to "Pacific Island countries and territories," with the abbreviation "PICs" retained consistently.

*Comment 2: Table 1 footnote* L272*.* We have placed the full terms for the flyway abbreviations (East Asian-Australasian Flyway, EAAF; West Pacific Ocean Flyway, WPOF) within the table cell rather than the footnote, as suggested.

*Comment 3: Duplicated citation [41].* The duplicated citation has been corrected to a single reference.

*Comment 4: Duplicated citation [54].* The duplicated citation has been corrected to a single reference.

#### Reviewer 2

*Comment: Keywords missing.* We confirm that keywords are included in the manuscript and have verified they are entered in the submission system.

We thank Reviewer 2 for their positive assessment.

#### Reviewer 3

**Results section structure**

*Comment: Results should begin with headline risk characterisation findings rather than the Baseline JEV Status section.* We appreciate this observation and agree that the integrated risk classification should be easy to locate and orient around from the outset. We have addressed this by adding a brief orienting paragraph at the opening of the Results section (L298-302) that previews the three risk profiles and their implications for surveillance before the detailed components are described. We hope this resolves the concern that the headline finding was not surfaced early enough.

We have nonetheless retained the existing sequence of components (Baseline JEV Status → Release → Exposure → Risk Characterisation) for the following reasons. This structure deliberately mirrors the logic of the risk analysis framework on which the assessment is built: release and exposure are assessed as independent components before being integrated into an overall risk characterisation. Presenting the synthesis (Table 4) before its inputs would ask readers to interpret the classification before seeing the evidence that underpins it. We note that the other two reviewers found this structure clear and logical. Regarding the Baseline JEV Status section specifically, we have retained this in the Results because the region's documented transmission history is essential context for interpreting the suitability assessment that follows. It establishes what is known about JEV in the Pacific and grounds the risk assessment in observed events. We are conscious, however, that this section is detailed, and the new orienting paragraph should help readers situate it within the broader structure.

**Editorial and data presentation modifications**

*Comment: Table 2 distance threshold.* We have added a footnote to Table 2 (L458) that explicitly defines the threshold criteria used for each release risk category (High: within EAAF and <1,000 km from a JEV-endemic country; Moderate: within the WPOF, or within EAAF and >1,000 km from a JEV-endemic country; Low: >1,000 km from an endemic country, outside EAAF and WPOF).

*Comment: Figure 4 — distinguish no-data countries from true-zero values* (L615). Marshall Islands and Pitcairn Islands, for which no pig density data were available, are now displayed in grey and distinguished from Guam, which has a true zero value. This distinction has also been clarified in the figure caption.

*Comment: Define PNG at first use.* Papua New Guinea is now defined in full at its first appearance in the Introduction, with the abbreviation PNG used thereafter.

*Comment: Define FAOSTAT at first use.* The Food and Agriculture Organization Corporate Statistical Database (FAOSTAT) is now defined at first use in the Methods section.

*Comment: Pig density and Low Risk classification.* We thank the reviewer for identifying this ambiguity. The role of pig density in the Low Risk classification has been clarified in both the Methods (L289-291) and Results sections (L639-651). Countries were classified as Low Risk where low vector diversity and low waterbird diversity were combined with low or moderate pig density — that is, pig density was a contributing criterion. We further clarified, with reference to Table 4 and its footnote, why countries such as Wallis and Futuna and Cook Islands were classified as Epidemic Potential despite low vector diversity: their very high pig densities (>190 pigs per 100 people), combined with multiple vector species, were judged sufficient to support epidemic amplification. By contrast, Niue and Tokelau, which also have high pig densities but only one or two vector species, were retained in the Low Risk category because the vector community was considered insufficient to support sustained transmission.

*Comment: Missing and misplaced parentheses.* We have reviewed and corrected the parentheses on the lines identified.

*Comment: Shorebird sentence — typo and ambiguity.* We have corrected the grammatical error and clarified that shorebirds have not been experimentally tested for their JEV host competence, rather than implying they have been studied and found negative.

*Comment: Typo, page 21.* The extraneous "with" has been removed.

*Comment: Three countries listed, only two named.* We thank the reviewer for identifying this omission. Tuvalu has been added to the list of PICs classified as moderate introduction risk due to WPOF connectivity, correcting the discrepancy between the count and the list.

*Comment: "Expended" should be "expanded," page 32.* This has been corrected.
