# Supplementary material for: Qualitative risk assessment of Japanese encephalitis virus introduction and transmission in Pacific Island countries and territories
Source: PLoS Negl Trop Dis. 2026 Sep 3;20(9):e0014162. doi: 10.1371/journal.pntd.0014162 (PMC13588533; doi:10.1371/journal.pntd.0014162)
Supplement: Peer review history

# Peer review: Decision Letter 0

**DOI:** 10.1371/journal.pntd.0014162.r001

**Authors:** Richard A. Bowen, Qu Cheng

11 Jun 2026

PNTD-D-26-00554

Qualitative risk assessment of Japanese encephalitis virus introduction and transmission in Pacific Island Countries and areas

PLOS Neglected Tropical Diseases

Dear Dr. Skinner,

Thank you for submitting your manuscript to PLOS Neglected Tropical Diseases. After careful consideration, we feel that it has merit but does not fully meet PLOS Neglected Tropical Diseases's publication criteria as it currently stands. Therefore, we invite you to submit a revised version of the manuscript that addresses the points raised during the review process.

Please submit your revised manuscript by Aug 10 2026 11:59PM. If you will need more time than this to complete your revisions, please reply to this message or contact the journal office at plosntds@plos.org. When you're ready to submit your revision, log on to https://www.editorialmanager.com/pntd/ and select the 'Submissions Needing Revision' folder to locate your manuscript file.

Please include the following items when submitting your revised manuscript:

* A letter that responds to each point raised by the editor and reviewer(s). You should upload this letter as a separate file labeled 'Response to Reviewers'. This file does not need to include responses to any formatting updates and technical items listed in the 'Journal Requirements' section below.  * A marked-up copy of your manuscript that highlights changes made to the original version. You should upload this as a separate file labeled 'Revised Manuscript with Track Changes'.* An unmarked version of your revised paper without tracked changes. You should upload this as a separate file labeled 'Manuscript'.If you would like to make changes to your financial disclosure, competing interests statement, or data availability statement, please make these updates within the submission form at the time of resubmission. Guidelines for resubmitting your figure files are available below the reviewer comments at the end of this letter.As the corresponding author, your ORCID iD is verified in the submission system and will appear in the published article. PLOS supports the use of ORCID, and we encourage all coauthors to register for an ORCID iD and use it as well. Please encourage your coauthors to verify their ORCID iD within the submission system before final acceptance, as unverified ORCID iDs will not appear in the published article. Only the individual author can complete the verification step; PLOS staff cannot verify ORCID iDs on behalf of authors.We look forward to receiving your revised manuscript.  Kind regards,

Richard A. Bowen, DVM PhD

Academic Editor PLOS Neglected Tropical Diseases Qu Cheng Section Editor PLOS Neglected Tropical Diseases

Shaden Kamhawi

co-Editor-in-Chief

PLOS Neglected Tropical Diseases

orcid.org/0000-0003-4304-636XX

Paul Brindley

co-Editor-in-Chief

PLOS Neglected Tropical Diseases

orcid.org/0000-0003-1765-0002

**Additional Editor Comments (if provided):**

Your manuscript has been reviewed by 3 experts, each of whom found it valuable and generally well written, but each as offered a few minor suggestions for improvement. Please evaluate these reviewer comments, modify your manuscript as you feel appropriate and resubmit along with a point-by-point response to reviewer comments.

**Journal Requirements:**

If the reviewer comments include a recommendation to cite specific previously published works, please review and evaluate these publications to determine whether they are relevant and should be cited. There is no requirement to cite these works unless the editor has indicated otherwise.

1) Please ensure that the CRediT author contributions listed for every co-author are completed accurately and in full.

At this stage, the following Authors/Authors require contributions: Amanda Murphy. Please ensure that the full contributions of each author are acknowledged in the "Add/Edit/Remove Authors" section of our submission form.

The list of CRediT author contributions may be found here: https://journals.plos.org/plosntds/s/authorship#loc-author-contributions

2) Please provide an Author Summary. This should appear in your manuscript between the Abstract (if applicable) and the Introduction, and should be 150-200 words long. The aim should be to make your findings accessible to a wide audience that includes both scientists and non-scientists. Sample summaries can be found on our website under Submission Guidelines:

https://journals.plos.org/plosntds/s/submission-guidelines#loc-parts-of-a-submission

3) Please upload all main figures as separate Figure files in .tif or .eps format. For more information about how to convert and format your figure files please see our guidelines:

https://journals.plos.org/plosntds/s/figures

4) Please upload a copy of Figure Fig1-4 which you refer to in your text on pages 20, 24, 29, and 30. Or, if the figure is no longer to be included as part of the submission please remove all reference to it within the text.

5) We have noticed that you have uploaded Supporting Information files, but you have not included a list of legends. Please add a full list of legends for your Supporting Information files after the references list.

6) Please amend your detailed Financial Disclosure statement. This is published with the article. It must therefore be completed in full sentences and contain the exact wording you wish to be published.

i) State what role the funders took in the study. If the funders had no role in your study, please state: "The funders had no role in study design, data collection and analysis, decision to publish, or preparation of the manuscript."

ii) If any authors received a salary from any of your funders, please state which authors and which funders..

If you did not receive any funding for this study, please simply state: u201cThe authors received no specific funding for this work.u201d

**Reviewers' comments:** Reviewer's Responses to Questions

**Key Review Criteria Required for Acceptance?**

As you describe the new analyses required for acceptance, please consider the following:

**Methods**

-Are the objectives of the study clearly articulated with a clear testable hypothesis stated?

-Is the study design appropriate to address the stated objectives?

-Is the population clearly described and appropriate for the hypothesis being tested?

-Is the sample size sufficient to ensure adequate power to address the hypothesis being tested?

-Were correct statistical analysis used to support conclusions?

-Are there concerns about ethical or regulatory requirements being met?

Reviewer #1: The methods section of the manuscript is well-structured, transparent, and appropriate for the study's objectives.

Objectives and hypothesis: The primary objective, to conduct a qualitative risk assessment of Japanese encephalitis virus (JEV) introduction and transmission across 22 Pacific Island Countries and areas (PICs), is explicitly and clearly articulated. The study aims to stratify these PICs into distinct risk profiles (endemic, epidemic, low potential) based on a multi-criteria assessment. While the study does not state a formal, testable hypothesis in the traditional sense, this is appropriate for a descriptive, risk-assessment framework where the goal is to synthesize existing evidence to characterize risk rather than to test a specific causal relationship. The research questions guiding the assessment are clear and logically flow from the introduction.

Study design: The choice of a qualitative risk assessment framework is a significant strength and is highly appropriate for addressing the stated objectives, particularly in a region where quantitative surveillance data on JEV are sparse or non-existent. The authors have adopted a standard, robust structure comprising a release assessment, an exposure assessment, and a risk characterization. This systematic approach enhances the rigor and reproducibility of the assessment. The use of a pre-specified, criteria-based scoring system for each component (e.g., vector suitability, host suitability, introduction risk) is commendable, as it provides a transparent and justifiable basis for the resulting classifications.

Population: The study population consists of all 22 PICs, which is clearly defined and comprehensive for a regional risk assessment. This inclusive approach ensures that the findings are relevant to the entire Pacific region and allows for comparative analysis between different island nations and territories. The data sources used to characterize these PICs, ranging from peer-reviewed literature and institutional databases (e.g., BirdLife International, FAOSTAT) to specific regional guides, are appropriate and well-documented.

Sample size: Traditional sample size and power calculations are not applicable to this study design. The authors conducted a comprehensive assessment of all 22 PICs, effectively performing a census of the region of interest rather than drawing a statistical sample. This approach is appropriate and ensures the completeness of the regional analysis.

Statistical analysis: The analysis is qualitative and descriptive, which is consistent with the study design. The authors use a classification system based on pre-defined, explicit thresholds (Table 1) to categorize risk components. The integration of these components to derive the final risk characterization is logical and clearly explained. The absence of complex statistical modeling is not a weakness but rather a reflection of the qualitative nature of the assessment and the limitations of the available data. The methods used are sufficient to support the study's conclusions.

Ethical/regulatory concerns: There are no concerns regarding ethical or regulatory requirements. The study is based entirely on the analysis of publicly available data, including published literature and aggregated data from public databases. No new data were collected from human or animal subjects, and therefore, institutional review board approval was not required.

Reviewer #2: Methodology is clear and reproducible.

Reviewer #3: The authors provide a nice summary of the three components in their assessment and do a nice job of defining key terms.

**********

**Results**

-Does the analysis presented match the analysis plan?

-Are the results clearly and completely presented?

-Are the figures (Tables, Images) of sufficient quality for clarity?

Reviewer #1: The results are presented in a clear, logical, and comprehensive manner that directly aligns with the methodology.

Analysis and presentation: The analysis presented in the results section perfectly matches the analysis plan outlined in the methods. The authors systematically present the findings for each component of the risk assessment: baseline JEV status, release assessment (introduction pathways), exposure assessment (vectors and hosts), and the final integrated risk characterization. This structure makes the results easy to follow and understand. The narrative is well-supported by quantitative details where available (e.g., seroprevalence percentages from historical studies, pig densities, number of vector species).

Clarity and completeness: The results are presented with a high degree of clarity and completeness. The "Baseline JEV status" section provides an excellent historical context that is crucial for interpreting the subsequent risk assessment. The detailed breakdown of vector species presence (Table 3) and host diversity (Figures 3 and 4) provides a rich dataset that underpins the final conclusions. The final synthesis in Table 4 is particularly effective, as it provides a clear, at-a-glance summary of the entire risk assessment for all 22 PICs, integrating multiple data streams into a coherent final classification. The reporting is robust and follows principles of good scientific communication.

Figures and tables: The figures and tables are of high quality, are directly relevant to the text, and significantly enhance the clarity of the results.

Reviewer #2: Yes, results are clear.

Reviewer #3: I got bogged down in the details under the Baseline JEV Status subheading. While the scoping review of baseline conditions is included in the Methods, the results of the scoping review feel out of place and, in my view, detract from the headline findings of the study (the *risk* assessment). I found such extensive description of the baseline conditions to be tangential to the question at hand, and suggest the authors begin the Results section with a high-level descriptive summary of which countries met which criteria under each assessment component and how they were classified overall. The visualization I was hoping to see reported first in the Results section appears later as Table 4. I understand why the authors chose to report the components in this order (Release --> Exposure --> Risk), though as a reader, I would encourage reorganizing the Results section to start with the headline result (Risk) followed by a more detailed unpacking of findings regarding the components of risk (Release and Exposure). The content under Baseline JEV status, in my view, could be streamlined substantially, moved to an appendix or possibly stand alone as its own paper.

**********

**Conclusions**

-Are the conclusions supported by the data presented?

-Are the limitations of analysis clearly described?

-Do the authors discuss how these data can be helpful to advance our understanding of the topic under study?

-Is public health relevance addressed?

Reviewer #1: The study’s conclusions are well supported by the data and carefully interpreted. It shows that 15 of 22 Pacific Island Countries (PICs) have ecological conditions suitable for JEV transmission, with clear classification into endemic, epidemic, and low-risk categories. A key strength is the authors’ transparent discussion of limitations, including data gaps, reliance on presence/absence information, and uncertainty in transmission dynamics. The study makes a significant contribution by providing the first comprehensive regional risk framework, moving beyond simple risk categories. It is highly relevant for public health, offering practical guidance for surveillance, preparedness, and response, especially through tailored strategies for endemic versus epidemic settings.

Reviewer #2: Yes.

Reviewer #3: The authors' conclusions are well supported by the data and analysis, as presented.

**********

**Editorial and Data Presentation Modifications?**

Use this section for editorial suggestions as well as relatively minor modifications of existing data that would enhance clarity. If the only modifications needed are minor and/or editorial, you may wish to recommend “Minor Revision” or “Accept”.

Reviewer #1: The manuscript is well-written, and only very minor editorial suggestions are needed. The authors should consider these minor points to further enhance clarity and consistency.

1. Terminology consistency: The manuscript alternates between "Pacific Island Countries and areas (PICs)" and "Pacific Island countries and territories." While the meaning is clear, selecting one term and using it consistently throughout the title, abstract, and main text would improve stylistic consistency.

2. Table 1 footnote: In Table 1, the footnote for the "Introduction risk" row defines "EAAF" and "WPOF." It might be slightly clearer to place the abbreviations in parentheses within the table itself, e.g., "East Asian-Australasian Flyway (EAAF)", and reserve the footnote for the explanatory note about upgrading ardeid suitability. This is a minor stylistic preference.

3. Line 218: The citation "[41][41]" appears to be a duplication. Please correct to a single citation.

4. Page 31: The citation "[54][54]" appears to be a duplication. Please correct to a single citation.

Reviewer #2: Keywords missing. Therefore, Minor revision is required.

Reviewer #3: In Table 2, there’s no indication of what the distance threshold is for distance to JEV endemic countries. To facilitate readers’ interpretation, I would suggest either revising the column of the table to indicate which category each country fell into, or including a reminder in the caption or column label what the threshold was.

In Figure 4, can the countries for which no data are available (Marshall Islands, Pitcairn Islands) be visually distinguished from the country that appears to have a true zero value for pig density (Guam)?

**********

**Summary and General Comments**

Use this section to provide overall comments, discuss strengths/weaknesses of the study, novelty, significance, general execution and scholarship. You may also include additional comments for the author, including concerns about dual publication, research ethics, or publication ethics. If requesting major revision, please articulate the new experiments that are needed.

Reviewer #1: This manuscript presents a timely, rigorous, and highly significant qualitative risk assessment of Japanese encephalitis virus in the Pacific Island Countries and areas. The study addresses a critical knowledge gap concerning the potential for JEV expansion into a region where surveillance and diagnostic capacity are limited. The recent emergence of JEV in mainland Australia makes this work particularly relevant and urgent.

Strengths:

1. Novelty and significance: This is the first comprehensive, systematic assessment of JEV risk across the entire Pacific region. Its findings provide a crucial foundation for public health preparedness and future research.

2. Methodological rigor: The use of a structured, transparent, and criteria-based qualitative framework is a major strength, lending credibility and reproducibility to the findings in a data-limited context.

3. Clarity and presentation: The manuscript is well-written and logically organized. The high-quality figures and tables effectively summarize complex information and strongly support the narrative.

4. Public health impact: The study has direct and immediate relevance for public health policy, surveillance design, and resource allocation in the Pacific. The distinction between endemic and epidemic risk profiles provides actionable guidance for national and regional health authorities.

5. Scholarly integrity: The authors provide a balanced perspective, including a thorough and honest discussion of the study's limitations, which strengthens the overall credibility of the work.

Weaknesses: The primary weaknesses of the study are inherent to the qualitative risk assessment approach and the available data, rather than flaws in the execution. The authors have done an outstanding job of identifying and discussing these limitations themselves, including the reliance on presence/absence data, uncertainties in introduction pathways, and the lack of subnational detail.

Overall recommendation: This is an excellent manuscript that represents a significant contribution to the fields of infectious disease epidemiology, veterinary public health, and global health security. It is well-executed, clearly presented, and has profound public health implications. I recommend this manuscript for publication in PLOS Neglected Tropical Diseases following minor revisions, and other minor editorial points noted above.

Reviewer #2: Great work authors.

Sorry for the delay.

Reviewer #3: This is an interesting analysis and well-written paper on the risk of introduction and transmission of Japanese encephalitis virus in the Pacific. The analysis is timely given the recent emergence of JEV in Australia, and the authors provide concrete and tractable recommendations in light of their findings. I find the manuscript worthy of publication with a few minor revisions, as described in more substantive comments above and minor/typographical comments below:

Define acronyms on first use. The acronym PNG is first used in the main text in line 124 (Introduction) but not defined until line 247 (Results). The full name of FAOSTAT is used in lines 210-211 but the acronym that is used in the next line is never defined.

In lines 238-240, the authors state, “Countries with low vector diversity and low waterbird diversity were classified as Low Risk, reflecting insufficient host-vector combinations to support sustained transmission cycles.” Did pig density not figure into this designation? If so, why?

Double-check manuscript for missing and misplaced parentheses (I noted some on lines 216, 255, caption of Figure 3)

Duplicated citation on line 218

In lines 352-353, the authors state, “However, this flyway mostly supports shorebirds which are have not been considered as JEV hosts, and only one documented ardeid species.” There is a typo in this sentence that requires clarification. Have the shorebirds in the flyway not been considered a JEV hosts (that is, they have not been studied at all)? Or have they been studied without evidence to date of JEV infection?

Line numbers were lost after line 445 (presumably due to the section break for Table 3’s landscape orientation)

Final line of page 21: typo, remove “with”

Double-check parenthetical references. On page 25, when the authors describe variation in introduction risk, they state “three countries… were classified as moderate introduction risk due to being within the WPOF” but only list two countries (Nauru and Federated States of Micronesia) in parentheses.

Typo on page 32, third sentence under “Future surveillance and research priorities”: expended should be expanded

**********

PLOS authors have the option to publish the peer review history of their article (what does this mean?). If published, this will include your full peer review and any attached files.

If you choose “no”, your identity will remain anonymous but your review may still be made public.

**Do you want your identity to be public for this peer review?**  For information about this choice, including consent withdrawal, please see our Privacy Policy.

Reviewer #1: No

Reviewer #2: **Yes:** DR. DENNY MATHEW JOHN

Department of Community Medicine

Kerala Medical College Hospital

Palakkad, Kerala, India

Reviewer #3: No

[NOTE: If reviewer comments were submitted as an attachment file, they will be attached to this email and accessible via the submission site. Please log into your account, locate the manuscript record, and check for the action link "View Attachments". If this link does not appear, there are no attachment files.] **Figure resubmission:**   While revising your submission, we strongly recommend that you use PLOS’s NAAS tool (https://ngplosjournals.pagemajik.ai/artanalysis) to test your figure files. NAAS can convert your figure files to the TIFF file type and meet basic requirements (such as print size, resolution), or provide you with a report on issues that do not meet our requirements and that NAAS cannot fix.

After uploading your figures to PLOS’s NAAS tool - https://ngplosjournals.pagemajik.ai/artanalysis, NAAS will process the files provided and display the results in the "Uploaded Files" section of the page as the processing is complete. If the uploaded figures meet our requirements (or NAAS is able to fix the files to meet our requirements), the figure will be marked as "fixed" above. If NAAS is unable to fix the files, a red "failed" label will appear above. When NAAS has confirmed that the figure files meet our requirements, please download the file via the download option, and include these NAAS processed figure files when submitting your revised manuscript. **Reproducibility:**  To enhance the reproducibility of your results, we recommend that authors of applicable studies deposit laboratory protocols in protocols.io, where a protocol can be assigned its own identifier (DOI) such that it can be cited independently in the future. Additionally, PLOS ONE offers an option to publish peer-reviewed clinical study protocols. Read more information on sharing protocols at https://plos.org/protocols?utm_medium=editorial-email&utm_source=authorletters&utm_campaign=protocols

# Author comment: Author response to Decision Letter 1

**DOI:** 10.1371/journal.pntd.0014162.r002

16 Aug 2026

Attachment Submitted filename: Skinner_etal_JEV PICs_ResponseToReviewers_UQ.docx

# Editor report: Decision Letter 1

**DOI:** 10.1371/journal.pntd.0014162.r003

**Authors:** Richard A. Bowen, Qu Cheng

20 Aug 2026

Dear Dr Skinner,

We are pleased to inform you that your manuscript 'Qualitative risk assessment of Japanese encephalitis virus introduction and transmission in Pacific Island countries and territories' has been provisionally accepted for publication in PLOS Neglected Tropical Diseases.

Before your manuscript can be formally accepted you will need to complete some formatting changes, which you will receive in a follow up email. A member of our team will be in touch with a set of requests.

Please note that your manuscript will not be scheduled for publication until you have made the required changes, so a swift response is appreciated.

IMPORTANT: The editorial review process is now complete. PLOS will only permit corrections to spelling, formatting or significant scientific errors from this point onwards. Requests for major changes, or any which affect the scientific understanding of your work, will cause delays to the publication date of your manuscript.

Should you, your institution's press office or the journal office choose to press release your paper, you will automatically be opted out of early publication. We ask that you notify us now if you or your institution is planning to press release the article. All press must be co-ordinated with PLOS.

Thank you again for supporting Open Access publishing; we are looking forward to publishing your work in PLOS Neglected Tropical Diseases.

Best regards,

Richard A. Bowen, DVM PhD

Academic Editor

PLOS Neglected Tropical Diseases

Qu Cheng

Section Editor

PLOS Neglected Tropical Diseases

Shaden Kamhawi

co-Editor-in-Chief

PLOS Neglected Tropical Diseases

orcid.org/0000-0003-4304-636XX

Paul Brindley

co-Editor-in-Chief

PLOS Neglected Tropical Diseases

orcid.org/0000-0003-1765-0002

***********************************************************

Thank you for the revisions to your manuscript, which correct some errors and clarify some points. This is a very interesting and valuable piece of work, acceptable for publication in PLoS NTD.

# Editor report: Acceptance letter

**DOI:** 10.1371/journal.pntd.0014162.r004

**Authors:** Richard A. Bowen, Qu Cheng

Dear Dr Skinner,

We are delighted to inform you that your manuscript, "Qualitative risk assessment of Japanese encephalitis virus introduction and transmission in Pacific Island countries and territories," has been formally accepted for publication in PLOS Neglected Tropical Diseases.

We have now passed your article onto the PLOS Production Department who will complete the rest of the publication process. All authors will receive a confirmation email upon publication.

The corresponding author will soon be receiving a typeset proof for review, to ensure errors have not been introduced during production. Please review the PDF proof of your manuscript carefully, as this is the last chance to correct any scientific or type-setting errors. Please note that major changes, or those which affect the scientific understanding of the work, will likely cause delays to the publication date of your manuscript. Note: Proofs for Front Matter articles (Editorial, Viewpoint, Symposium, Review, etc...) are generated on a different schedule and may not be made available as quickly.

Soon after your final files are uploaded, the early version of your manuscript will be published online unless you opted out of this process. The date of the early version will be your article's publication date. The final article will be published to the same URL, and all versions of the paper will be accessible to readers.

For Research Articles, you will receive an invoice from PLOS for your publication fee after your manuscript has reached the completed accept phase. If you receive an email requesting payment before acceptance or for any other service, this may be a phishing scheme. Learn how to identify phishing emails and protect your accounts at https://explore.plos.org/phishing.

Thank you again for supporting open-access publishing; we are looking forward to publishing your work in PLOS Neglected Tropical Diseases.

Best regards,

Shaden Kamhawi

co-Editor-in-Chief

PLOS Neglected Tropical Diseases

Paul Brindley

co-Editor-in-Chief

PLOS Neglected Tropical Diseases
